# Isoginkgetin increases the expression of metal responsive transcripts but inhibits their translation

**DOI:** 10.1371/journal.pone.0352014

**Published:** 2026-06-25

**Authors:** Claire Peneycad, Erin van Zyl, Abhinav Mohur, Bruce C. McKay

**Affiliations:** 1 Department of Biology, Carleton University, Ottawa, Ontario, Canada; 2 Institute of Biochemistry, Carleton University, Ottawa, Ontario, Canada; Public Library of Science, UNITED KINGDOM OF GREAT BRITAIN AND NORTHERN IRELAND

## Abstract

Isoginkgetin (IGG) is a natural bioflavonoid isolated initially from leaf extracts of *Gingko biloba* trees used in traditional Chinese medicine. We previously reported that IGG strongly inhibits nascent protein synthesis by activating the shared ATF4-dependent branches of the integrated stress response (ISR) and the unfolded protein response (UPR). Here we sought to characterize an ATF4-independent response to IGG that leads to increased expression of mRNAs encoding metallothionines (MTs) and a zinc transporter (ZnT1). We confirm that IGG induces MT1F, MT1X, MT2A and ZnT1 mRNAs in several independent cell lines. We also find that siRNA-mediated knockdown of the metal regulatory transcription factor 1 (MTF-1) reduced basal and IGG-induced levels of these mRNAs. Curiously, we did not detect increased expression of these proteins following exposure to IGG. To study the dissociation between transcription and translation of metal responsive mRNAs, we created a stable cell line expressing a luciferase reporter gene under control of five metal response elements. Our positive control (ZnSO_4_) increased luciferase activity as expected but IGG reduced luciferase activity. Importantly, IGG, and other inhibitors of translation, prevented the increase in luciferase activity when combined with ZnSO_4_ without affecting the ZnSO_4_-induced increase in luciferase mRNA levels. We propose that IGG activates ATF4- and MTF-1-dependent transcriptional responses but that IGG simultaneously impairs nascent protein synthesis and masks the metal response at the protein level.

## Introduction

Isoginkgetin (IGG) is a natural bioflavonoid derived from *Ginkgo biloba* trees and abundant in *Ginkgo biloba* extracts [1]. These extracts have long been used in traditional Chinese medicine for their anti-inflammatory properties [1,2]. In 2008, IGG was identified in a chemical screen as a potent small molecule inhibitor of the spliceosome [2]. Spliceosomes are large ribonucleoprotein complexes that assemble in a stepwise manner along pre-mRNA transcripts at each intron [3] and catalyze the splicing reactions that remove introns and ligate exons [4]. A failure to properly splice pre-mRNAs could lead to either a failure to express proteins or the synthesis of abnormal proteins with altered function [5]. In this way, splicing defects could have grave effects on protein expression and function with critical consequences for cell function and organismal health [5].

Recently, we reported that IGG induces three specific stress-response pathways: the unfolded protein response (UPR), the integrated stress response (ISR) and the response to metal ions (RMI) [6–8]. The UPR and ISR are overlapping pathways that can involve the protein kinase R (PKR)-like endoplasmic reticulum kinase (PERK) leading to the phosphorylation of eIF2α, inhibition of cap-dependent translation and the selective activation of the activating transcription factor 4 (ATF4) transcription factor [1,9]. Consistent with this well characterized pathway, we found that IGG inhibited protein synthesis while permitting the selective translation of ATF4 and several of its known target genes [6]. RNA sequencing analysis led to the identification of 63 induced and 5 inhibited transcripts that exhibited an ATF4-dependent pattern of expression upon IGG exposure [8]. Eighteen of the 63 induced transcripts were known targets of ATF4 [6,10]. Therefore, the transcriptional response to IGG is mediated in large part through ATF4 [6–8]. Despite this, microarray and RNA-sequencing analyses both indicated that IGG also increased the expression of 5 metal responsive transcripts and these were induced independent of ATF4 [6–8].

Here we used independent methods to confirm that ATF4 was not required for IGG to induce these metal responsive transcripts. Instead, we found that the metal-responsive transcription factor 1 (MTF-1) was required to induce these transcripts in response to IGG. Surprisingly, the induction of metal responsive transcripts was not detected at the protein level. A disconnect between mRNA and protein expression was also detected using a metal responsive luciferase reporter gene. In fact, IGG prevented the increase in MTF-1-dependent luciferase activity following exposure to our heavy metal control (ZnSO_4_). The present work supports a role for MTF-1 in eliciting an IGG transcriptional response without increased expression of the encoded proteins, consistent with the inhibition of nascent protein synthesis by IGG. The present findings highlight the complexity of the cellular response to IGG with the UPR/ISR inhibiting the response to metal ions.

## Materials and methods

### Cell culture and drug treatment

HCT116, HeLa and U-2 OS cells were obtained from the American Tissue Type Collection (Cat #: CCL-247, CCL-2, and HTB-96, Manassas, VA). An HCT116-derived subline (HCT116 ATF4-def) that does not express ATF4 was previously generated in our lab using a tandem guide CRISPR-Cas9 strategy [8]. Normal human neonatal foreskin fibroblasts expressing human telomerase reverse transcriptase (NFhTERT) were obtained from Mats Ljungman (University of Michigan) [11]. HCT116, HCT116 ATF4-def, HCT116-XBP1mNG, and HCT116-ATF4mSC cells were grown in McCoys growth media (Multicell Wisent), supplemented with 9% mixture (1:3) of Newborn to Fetal Calf Serum (Multicell Wisent), 3% Fetal Bovine Serum (FBS) (Multicell Wisent), and 90 units/mL penicillin, and 90 ug/mL streptomycin (Multicell Wisent). HeLa, NFhTERT and U-2 OS cells were grown in Dulbecco’s Modified Eagle Medium (DMEM) High glucose (Multicell Wisent). HeLa DMEM growth medium was supplemented with 9% mixture (1:3) of Newborn to Fetal Calf Serum (Multicell Wisent), 3% Fetal Bovine Serum (FBS) (Multicell Wisent), and 90 units/mL penicillin, and 90 ug/mL streptomycin (Multicell Wisent). NFhTERT and U-2 OS DMEM growth medium was supplemented with 10% Fetal Bovine Serum (FBS) and 90 units/mL penicillin, and 90 ug/mL streptomycin (Multicell Wisent). For all experiments, cells were seeded a minimum of 16 hours prior to treatment at 5.0 × 10^6^ in 6-well dishes (HCT116, HCT116 ATF4-def, and NFhTERT) or 3.0 × 10^6^ in 6-well dishes (U-2 OS and HeLa). Cells were treated in growth media with 30 μM IGG (Calbiochem), 2 μM thapsigargin (Tg) (Millipore Sigma), 100 μM ZnSO_4_ (Millipore Sigma), 32 uM cycloheximide (CHX) (Millipore Sigma), or PERK inhibitor 1 (GSK2606414, abbreviated PERKi in the text) (Calbiochem). An equivalent volume of dimethyl sulfoxide (DMSO) (Calbiochem) was used as a vehicle control for IGG, Tg, and CHX. ZnSO_4_ stock solution was aqueous, so did not require a specific vehicle control.

### Reverse transcription (RT) and real time quantitative polymerase chain reaction (RT-qPCR)

At the time of collection, medium was removed, and cell monolayers were washed twice with PBS (pH 7.4). RNA was isolated using the EZ-10 DNA away RNA isolation kit (Bio Basic Canada) according to the manufacturer’s protocol. RNA concentration and quality was measured using a DeNovix DS-11 Spectrophotometer (DeNovix) and equal amounts of RNA were converted to cDNA using the High-capacity cDNA Reverse Transcription Kit (Applied Biosystems, ThermoFisher Scientific). cDNA was diluted with 200 μL RNAse free dH_2_O and RT-qPCR was completed with either the Bioline SensiFAST^TM^ SYBR HI-ROX Kit (FroggaBio Inc) or Bioline SensiFAST^TM^ SYBR NO-ROX Kit (FroggaBio Inc), with the following primers: MT2A F(GCACCTCCTGCAAGAAAAGCTG), R(CGGTCACGGTCAGGGTTGTA), MT1F F(CTGCTTCTTCGCTTCTCTCTT), R(ATCCAGGTTTGTACATGTCTCTC), MT1X F(TTTCCTCTTGATCGGGAACTC), R(CAAACGGGTCAGGGTTGTA), ZnT1 F (GAAGTGGTGATACAGTGGAAGT), R(TGTTAAGTTGTCCAGCCCTATC), MTF-1 F(CATTGGTCACTCTCCTGTTCTC), R(CTGGAAACCTCCTGCCTTATC), Firefly Luciferase F(GTGGTGTGCAGCGAGAATAG), R(CGCTCGTTGTAGATGTCGTTAG), and GAPDH F(GATTCCACCCATGGCAAATTC), R(GTCATGAGTCCTTCCACGATAC) which were used as a loading control. These primers were designed using either Primer–BLAST (NCBI) or Integrated DNA Technologies (IDT) design tool. All primers were synthesized by IDT (Ottawa, Ontario, Canada).

### Immunoblotting

Following treatment, cell monolayers were washed twice with PBS (pH 7.4) and collected in 1% SDS supplemented with a protease inhibitor tablet (Millipore Sigma). Cells were sonicated in 3 second pulses three times and remained on ice throughout. Protein was quantified using the Bio-Rad protein assay (Bio-Rad) and samples were prepared using NuPAGE LDS Sample buffer (4X) and DTT Sample reducing agent (10X) (ThermoFisher Scientific) for denaturing gel electrophoresis and protein reduction, respectively. Once prepared, even amounts of protein were loaded onto a NuPage 4−12% Bis-Tris gel (ThermoFisher Scientific) to detect MTF-1 and ZnT1. Due to their small size MTs were resolved on 16% NuPage Bis-Tris gels. Separated proteins were transferred to a nitrocellulose membrane which were then stained with Ponceau S (1.3 mg/mL in 1% acetic acid) to confirm equal loading and even transfer. Membranes were blocked in 5% Milk in TBST at room temperature for at least 1 hour. Membranes were then incubated in primary antibody: anti-ZnT1 (ThermoFisher Scientific, Cat. A305-424A), anti-MTF-1 (Abcam, Cat. AB236401), anti-MT (Abcam, Cat AB12228) or anti-Actin (Millipore Sigma, Cat. A5316) overnight at 4 °C. Following incubation, membranes were washed 4 times for 5 minutes in TBST (pH 7.6) and incubated with secondary antibody: 1:1000 (goat anti-mouse HRP conjugated, Abcam) or 1:2000 (goat anti-rabbit HRP conjugated, Abcam) for 2 hours at room temperature. Membranes were washed 2 times for 5 minutes and 2 times for 10 minutes in TBST. The membranes were then incubated in Clarity Western ECL substrate (Biorad) and imaged and quantified using the Fusion FX Viber Lourmat imager and VisionCap software.

### Disruption of MTF-1 with RNA interference

SMARTPool ON-TARGETplus Human MTF-1 siRNA and ON-TARGETplus Non-targeting Control Pool siRNAs (Horizon Discovery, Catalog ID: L-020078-00-0005 and D-001810-10-05) were prepared in 1X siRNA buffer according to the manufacturer’s instructions. U-2 OS cells were seeded in 6 cm dishes at 150,000 cells per dish in antibiotic free media 24 hours prior to transfection. Both siRNAs and the DharmaFECT 2 Transfection Reagent (Horizon Discovery) were then diluted in serum and antibiotic free media based on manufacturer’s recommendations and each diluted siRNA was combined with diluted transfection reagent to a final concentration of 50 nm. U-2 OS cells were then incubated in the combined transfection medium for 40 hours. Following transfection, U-2 OS cells were left in regular media for 24 hours, after which they were seeded in 6-well dishes. Cells were treated 24 hours later after which RNA was collected for analysis as previously described.

### Cellular localization of MTF-1

U-2 OS cells were seeded on sterilized poly-l-lysine-coated 22 mm square coverslips in a 6-well plate at 300,000 cells per well. Following treatment, cell monolayers were washed twice with PBS (pH 7.4). Cells were fixed in 3% paraformaldehyde in PBS for 15 minutes at room temperature and then washed twice in PBS for 5 minutes. Cells were permeabilized with 0.5% Triton X-100 in PBS for 6 minutes and then washed twice in PBS for 5 minutes. Cells were then blocked in 1% BSA in PBS for 2 hours at room temperature and then washed once in PBS. Cells were then incubated in primary antibody targeting MTF-1 (1:400 in 1% BSA in PBS, Abcam, Cat. Ab183897) and left overnight at 4°C. Following incubation, cells were washed 3 times for 5 minutes in PBS and then incubated in Alexa Fluor 488 secondary antibody (1:1000 in 1% BSA in PBS, ThermoFisher Scientific) for 1 hour at room temperature in the dark. Following incubation, cells were washed in PBS 3 times for 5 minutes in the dark. Following washes, coverslips were mounted onto microscope slides using VECTASHIELD Hardset Antifade Mounting Medium with DAPI (Vector Laboratories) and imaged using the Axio Observer 7 microscope using Zen Blue 2.6 software (Carl Zeiss, Oberkochen, Germany). Fluorescence was measured and analyzed using ImageJ software (National Institutes of Health, Maryland, USA).

### Generation of U-2 OS metal response element reporter cell line

To generate a metal response reporter cell line, we obtained the pGL4.40[luc2P/MRE/Hygro] Vector DNA (Promega, Catalog ID: E413A) in which luciferase expression in control by 5 copies of a metal response element (MRE). U-2 OS cells were seeded in a 96-well plate at 20,000 cells per well 24 hours prior to transfection. The Lipofectamine^**®**^ LTX Reagent (ThermoFisher Scientific, Cat: 15338030) was diluted in serum and antibiotic free DMEM medium according to the manufacturer’s instructions. Separately, the pGL4.40[luc2P/MRE/Hygro] Vector DNA was diluted in serum and antibiotic free DMEM media and Lipofectamine PLUS^TM^ Reagent (ThermoFisher Scientific, Cat: 15338030), according to the manufacturer’s instructions. The diluted DNA was combined with the diluted transfection reagent to a final concentration of 1 ng/uL. U-2 OS cells were then incubated in the combined transfection medium for 48 hours. Following transfection, U-2 OS cells were moved to a 24-well dish and grown in selection media with 100 ug/mL Hygromycin B (Millipore Sigma, Catalog: 10843555001). Successfully transfected cells were continuously cultured in hygromycin B selection media for further experimentation.

### Luciferase assay

U-2 OS MRE luciferase reporter cells were seeded in a 96-well plate at 15,000 cells per well 24 hours prior to treatment in phenol red-free DMEM media. Following treatment, a volume of the ONE-Glo^TM^ EX Reagent (Promega, Catalog: E8110) equal to the volume of treatment media was directly added to each well, according to the manufacturer’s instructions. Samples were incubated for 3 minutes on an orbital shaker at room temperature, allowing the reagent to lyse the cells. Following incubation, luminescence of samples was measured using the BioTek Cytation 5 Cell Imaging Multimode Reader (Agilent, Santa Clara, CA, USA).

### Nascent protein synthesis assay

A Click-iT® HPG Alexa Fluor® Protein Synthesis Assay (Invitrogen) was used to measure nascent protein synthesis [7]. Briefly, cells were seeded in 12 well dishes at 75, 000 cells per well, 24 hours before treatment. Cells were incubated with the indicated drug (IGG, TG, or CHX) in fresh growth medium for 23.5 hours. Medium was removed, and cells were incubated an additional 30 min with the same drugs along with 50 μM Click-iT® HPG in L-methionine-free medium to label nascent protein. Cells were then rinsed in PBS and fixed with 3.7% formaldehyde in PBS for 15 min. Cells were rinsed twice with 3% BSA in PBS, and permeabilized in 0.5% Triton® X-100 in PBS for 20 min. The permeabilization buffer was removed, cells were washed with 3% BSA in PBS and incubated with the Click-iT® reaction cocktail for 30 min at room temperature in the dark. Click-iT® reaction rinse buffer was used to wash cells once, followed by 2 washes in PBS. Cells were resuspended in PBS and analyzed on a per cell basis using a BD Accuri C6 flow cytometer, and BD Biosciences Accuri C6 software (BD Biosciences, Mississauga, ON).

## Results

### IGG induces metal responsive transcripts in an ATF4-independent manner

**Fig 1:**
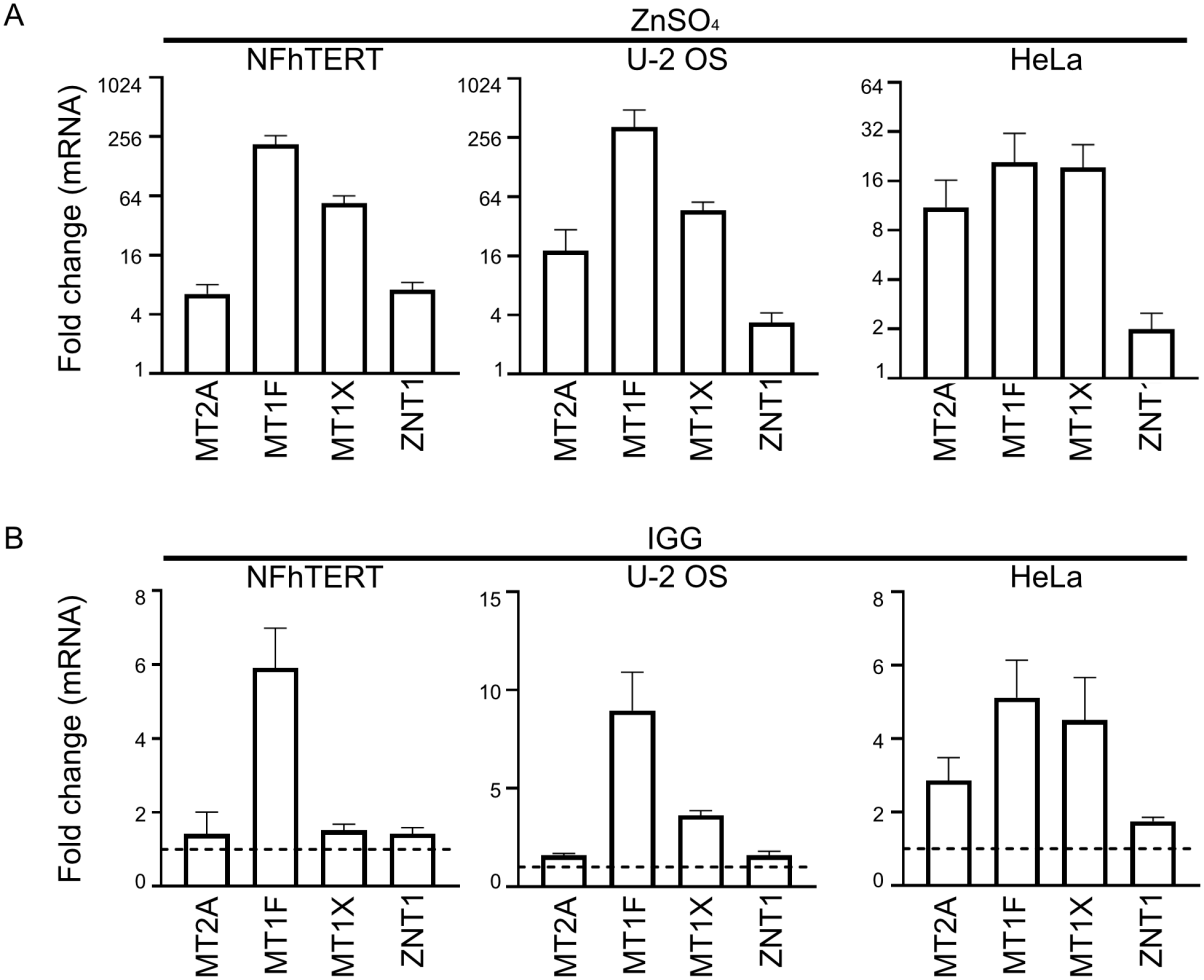
IGG increases expression of metal responsive transcripts. NFhTERT, U2-OS, and HeLa cells were treated with either ZnSO_4_ **(A)** or IGG **(B)** and RNA was collected 8 hours later for RT-qPCR analysis. Each value represents the mean fold change (+/- SEM) relative to its corresponding solvent control (aqueous or DMSO, respectively), determined from at least 4 independent experiments. Raw data is available in S1 File.

We have previously reported that IGG increased the expression of 4 metallothionines and a metal responsive zinc transporter in HCT116 colon cancer cells [6,8]. While the predominant transcriptional response involved the ATF4 transcription factor, this metal response appeared to be ATF4-independent in our RNA sequencing (RNA-seq) data set [6]. Here we sought to ensure that this IGG-induced response was not unique to the HCT116 cell line used. First, we tested human telomerase immortalized fibroblasts (NFhTERT), osteosarcoma cells (U-2 OS), and cervical cancer cells (HeLa) to ensure they had an intact response to metal ions following treatment with ZnSO_4_ (Fig 1A). The fold increase in expression of each metal responsive transcript exhibited a similar pattern across these transcripts in all 3 cells lines (MT1F>MT1X > MT2A>ZnT1) (Fig 1A). Treatment of these cell lines with IGG increased the expression of these transcripts but the fold change in expression was far lower than that induced by ZnSO_4_ and this varied by cell line (compare Fig 1A and B). For example, MT1F exhibited the largest fold increase in expression in response to ZnSO_4_ (250 versus 8-fold following ZnSO_4_ and IGG, respectively). Nonetheless, the underlying relationship among transcripts was similar (MT1F>MT1X > MT2A>ZnT1) (Fig 1B). Taken together, IGG significantly increased some metal responsive transcripts but this response was more modest than the ZnSO_4_ response.

**Fig 2:**
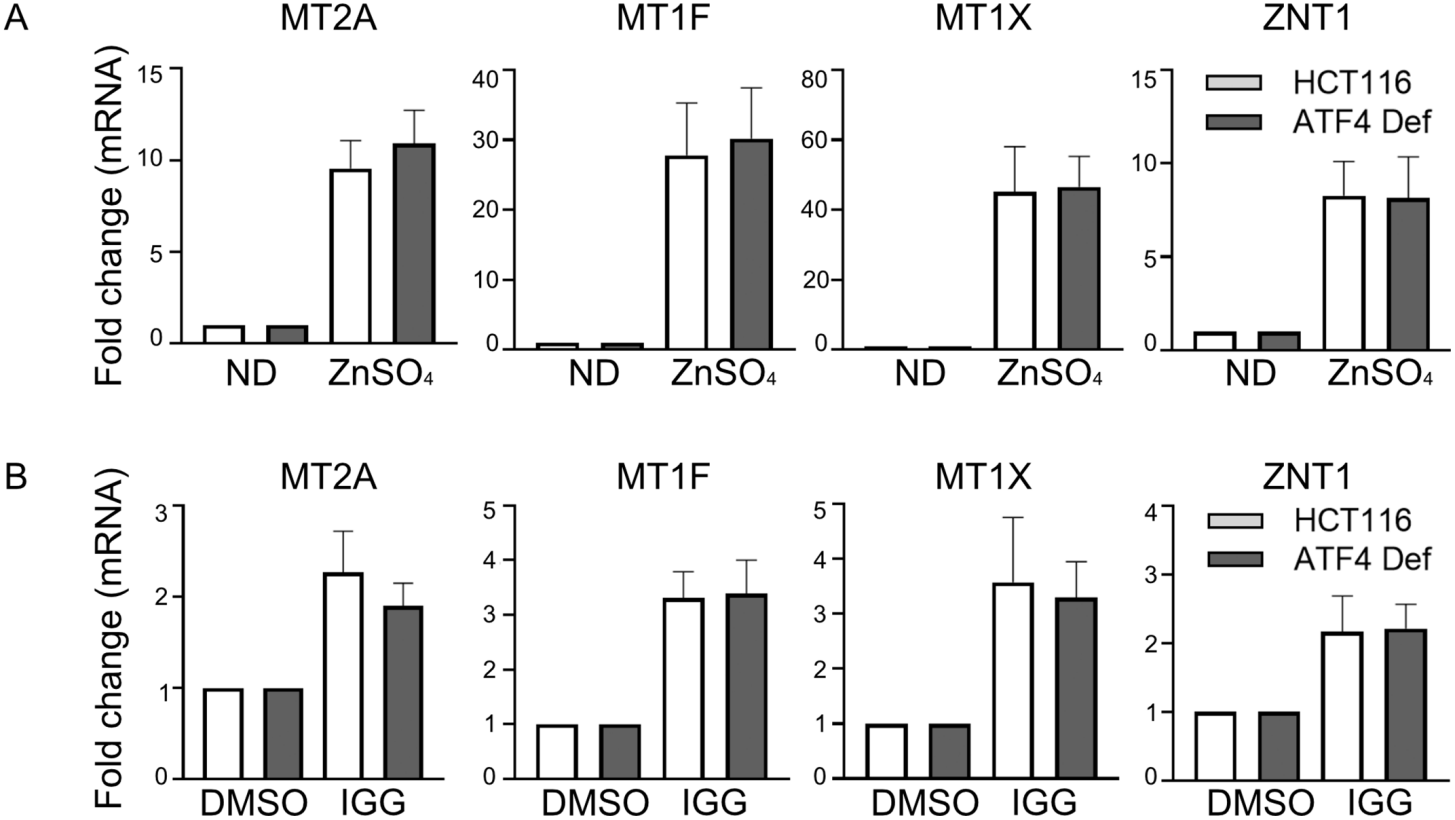
IGG induced metal responsive transcripts in an ATF4-independent manner. HCT116 and an isogenic ATF4-def subline were treated with ZnSO_4_ **(A)** or IGG **(B)** for 8 hours, and RNA was collected for RT-qPCR analysis. Each value represents the mean fold change (+/- SEM) relative to the corresponding control (untreated or DMSO vehicle control), determined from at least 5 independent experiments. Raw data is available in S2 File.

Our previous work suggested that ATF4 was central to the transcriptional response to IGG [6,8]. Therefore, we sought to determine the effect of ATF4 on IGG-induced expression of metal responsive transcripts using HCT116 cells and an isogenic ATF4-deficient subline (ATF4-def cells) created in our lab using CRISPR-Cas9 [6,8] (Fig 2A and 2B). MT2A, MT1F, MT1X and ZnT1 mRNA expression increased in both cell lines in response to our positive control ZnSO_4_, with the largest increases occurring in MT1F and MT1X again (recall Fig 1A). Importantly, IGG induced all four transcripts to a similar extent in both cell lines, consistent with our published RNA-seq data [6]. Taken together IGG leads to increased expression of these metal responsive transcripts in an ATF4-independent manner.

### Knockdown of MTF-1 reduces MT and ZnT1 mRNA expression in response to IGG

**Fig 3:**
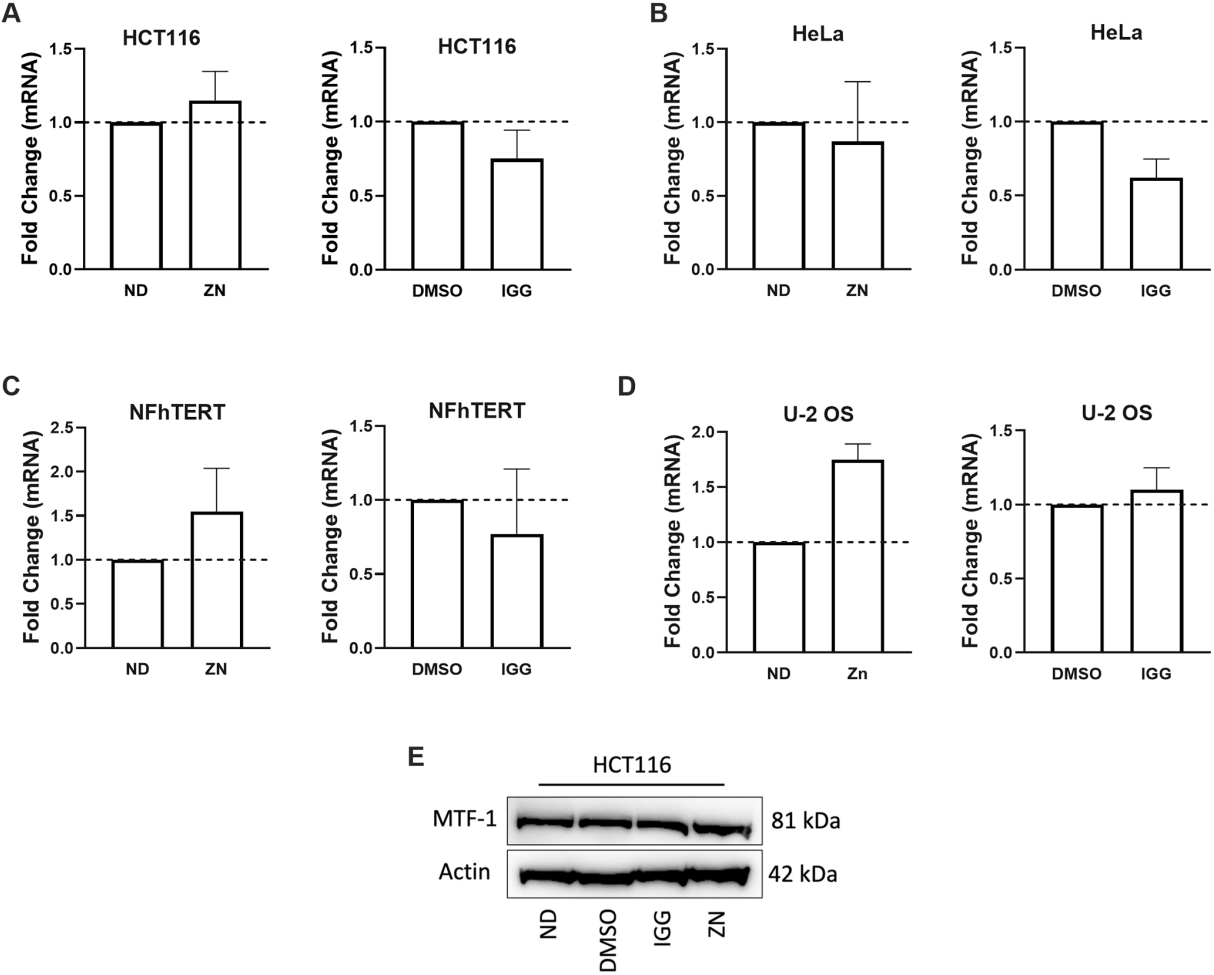
IGG does not increase MTF1 expression. HCT116 **(A and E)**, HeLa **(B)**, NFhTERT **(C)**, or U-2 OS **(D)** cells were treated with ZnSO_4_ or IGG for 8 hours. RNA and protein were collected for RT-qPCR (A-D) and immunoblot analysis **(E)**. Each value in A-D represents the mean fold change (+/- SEM) determined from a minimum of 3 independent experiments. The immunoblot presented in **(E)** is consistent with MTF1 expression detected in 3 separate immunoblot experiments. Uncropped immunoblots are provided as S1 Fig. Raw data is available in S3 File.

Metal responsive gene expression is largely dependent on the MTF-1 transcription factor [12,13]. To investigate the effects of IGG on MTF-1, mRNA expression was measured by RT-qPCR in various cell lines to determine if MTF-1 is regulated in response to IGG. MTF-1 was not induced by IGG in any cell line tested (Fig 3A–3D). Furthermore, ZnSO_4_ did not significantly induce MTF-1 mRNA expression in 3 of the 4 cell lines, with only a small increase in U-2 OS cells (Fig 3A–3D). MTF-1 was also assessed by immunoblot analysis and MTF-1 levels remained unaltered by IGG and ZnSO_4_ (Fig 3E). Thus, the current data indicates that MTF-1 mRNA and protein expression are not induced in response to IGG or ZnSO_4_.

**Fig 4:**
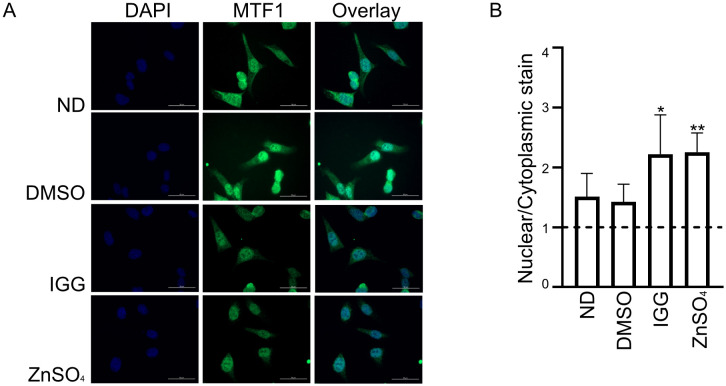
IGG appears to induce a small increase in the localization of MTF-1 to the nucleus. **(A)** U-2 OS cells were treated with IGG or ZnSO_4_ for 8 hours. Representative immunofluorescent staining of MTF-1 is presented from one of three independent experiments. **(B)** Integrated density was quantified using ImageJ software to compare MTF-1 nuclear fluorescence as a ratio to that in the cytoplasm. Each value represents the mean (+ SEM) determined from 5 independent experiments. * or ** indicates that the indicated value is significantly different (P < 0.05, and P < 0.01, respectively) from the value one, determined by a one-sample t-test. Raw data is available in S4 File.

This was not surprising because MTF-1 DNA binding activity is regulated in large part through subcellular localization [14,15]. Upon activation by various stressors, the proportion of MTF-1 in the nucleus increases [14,15]. We performed immunofluorescence microscopy in U-2 OS cells to visualize the subcellular localization of MTF-1 between the nucleus and cytoplasm to determine if IGG could induce the subcellular redistribution of MTF-1 (Fig 4A and 4B). In the untreated control cells, MTF-1 (shown in green) appears evenly distributed throughout the cells, with immunostaining in both the cytoplasm and nucleus (Fig 4A). In the cells treated with ZnSO_4_, the cytoplasmic staining was reduced compared to the nucleus. In the cells treated with IGG, there also seems to be an increase of MTF-1 within the nucleus compared to the untreated controls, but this change seemed less pronounced than the positive control, ZnSO_4,_ suggesting that IGG does not induce activation of MTF-1 as strongly as ZnSO_4_.

This analysis is subjective, so the relative intensity of cytoplasmic and nuclear MTF-1 staining was quantified from multiple images obtained from several independent experiments using ImageJ software. Integrated density was measured in the cytoplasm and nucleus, and nuclear staining is expressed relative to cytoplasmic staining intensity (Fig 4B). There appeared to be slightly more nuclear staining prior to treatment, although this was not statistically significant. Following treatment with either IGG or ZnSO_4_, there was a small increase in nuclear stain relative to cytoplasmic staining. Although the ratio of nuclear to cytoplasmic MTF-1 was greater than 1 in IGG and ZnSO_4_ treated samples, there was no significant difference in these ratios among the four conditions (P > 0.05 by One Way ANOVA). These findings suggest that there could be a small shift in the proportion of MTF-1 from the cytoplasm to the nucleus, but it remains equivocal.

**Fig 5:**
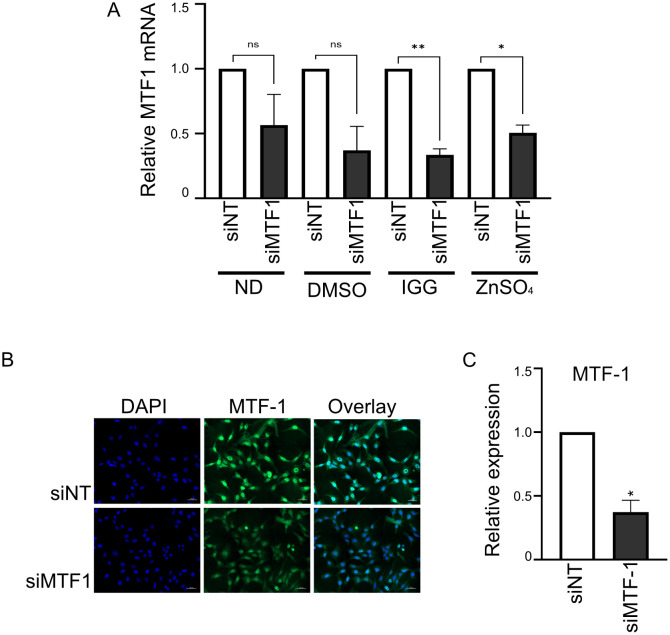
Efficacy of siRNAs targeting MTF-1 in U-2 OS cells. Cells were transfected with control non-targeting or MTF-1-targeting siRNAs for 40 hours. Transfected cells were subsequently exposed to the indicated compounds (ND indicates there was no drug treatment). **(A)** Eight hours following treatment, RNA samples were collected for RT-qPCR analysis. **(B)** Similarly treated cells underwent immunofluorescence analysis of MTF-1 expression **(B)**. **(C)** Relative MTF-1 levels were estimated from 3 independent experiments using ImageJ software. Values in A and C represent the mean (+ SEM). * and ** indicate that the mean is not equal to the value one (P < 0.05 or <0.01, respectively by a one-sample t-test).

To determine more definitively if MTF-1 contributes to MT and ZnT1 expression following IGG, we used an RNA interference approach to reduce MTF-1 levels in U-2 OS cells. MTF-1 targeting small interfering RNAs (siRNAs) were transfected into U-2 OS cells and MTF-1 expression was compared to control non-targeting siRNAs. RT-qPCR analysis indicated that MTF-1 levels decreased 2-fold or more in MTF-1 siRNA transfected cells, regardless of drug treatment (Fig 5A). Similarly, immunofluorescence microscopy indicated that siRNAs targeting MTF-1 led to a similar decrease in MTF-1 protein levels (Fig 5B and 5C), indicating that MTF-1 levels were significantly reduced following siRNA transfection.

**Fig 6:**
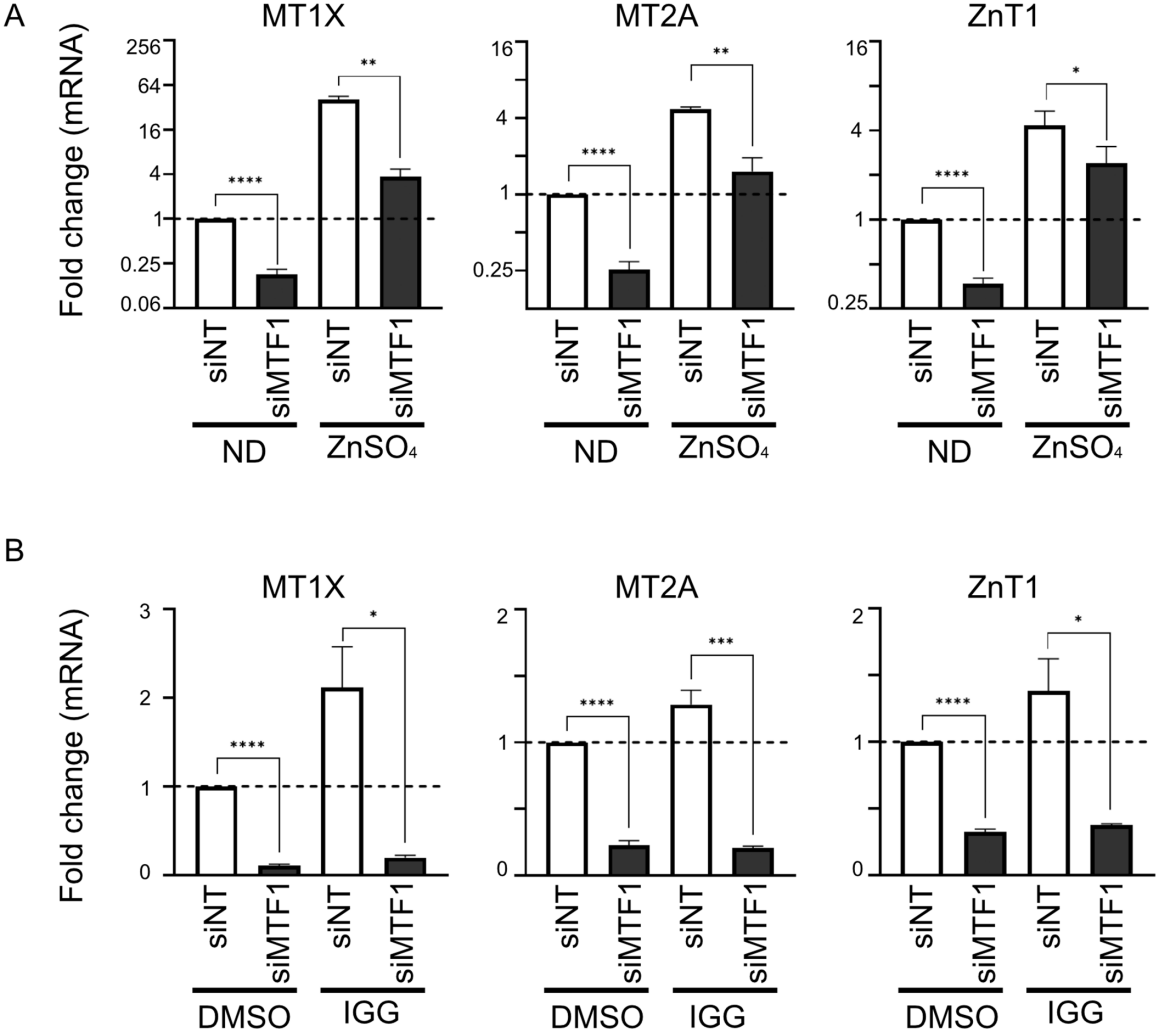
MTF-1 siRNAs reduce MT and ZnT1 mRNA expression. U-2 OS cells were transfected with control non-targeting or MTF-1-targeting siRNAs for 40 hours. These cells were treated with no drug (ND), DMSO, ZnSO4 **(A) or** IGG **(B)** for 8 hours and RT-qPCR analysis was performed for MT1X, MT2A and ZnT1 mRNA expression. Each value represents the mean fold change relative to the corresponding control, determined from at least 3 independent experiments. *, **, ***, and **** indicates that the indicated value is significantly different (P < 0.05, P < 0.01, P < 0.005, and P < 0.001, respectively) from its corresponding non-targeting siRNA transfected control sample, determined by unpaired t-tests. Raw data is available in S5 File.

Knockdown of MTF-1 resulted in a noticeable reduction in the expression of the MT1X, MT2A and ZnT1 mRNAs in untreated and solvent controls (Fig 6A and 6B). ZnSO_4_ strongly induced expression of all target mRNAs in the control siRNA transfected cells, but knockdown of MTF-1 prevented the full induction of these mRNAs by ZnSO_4_ (Fig 6A). IGG led to small increases in target mRNA expression in control siRNA transfected cells while IGG failed to induce MT1X, MT2A and ZnT1 mRNA to control levels in MTF-1 knockdowns (Fig 6B). The fact that siRNAs resulted in modest decreases in MTF-1 expression suggests that these experiments likely underestimate the contribution of MTF-1 to metal responsive mRNA expression. Overall, the data suggest that MTF-1 contributes strongly to the basal levels of MT1X, MT2A and ZnT1 mRNA in these cells and that MTF-1 is also required for the full induction of these mRNAs in response to either IGG or ZnSO_4_.

### ZnSO_4_ induces metal responsive mRNAs and proteins while IGG only induces the mRNA

**Fig 7:**
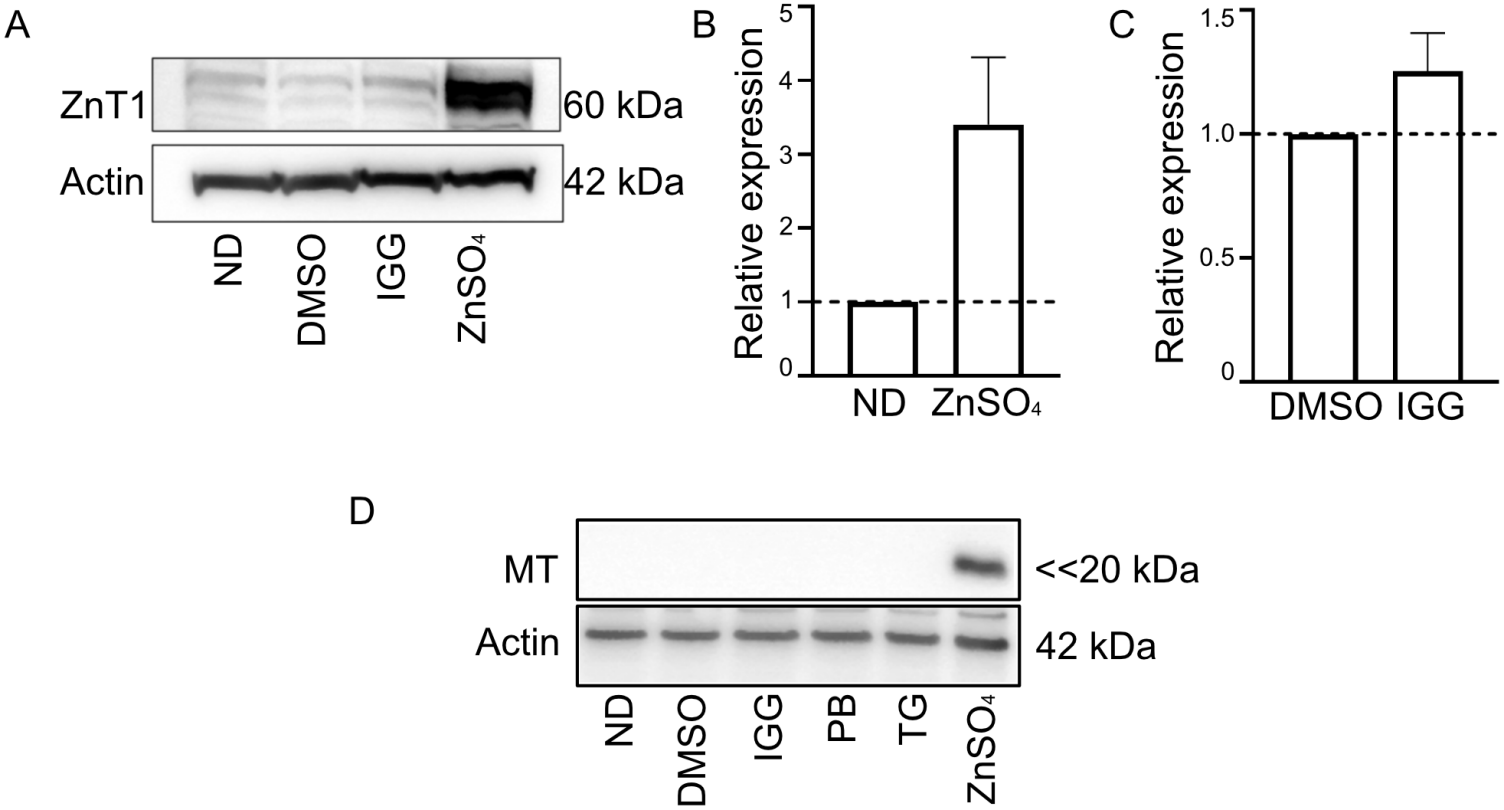
IGG does not induce metal responsive protein expression. **(A)** HCT116 cells were treated with IGG, or ZnSO_4_ for 8 hours and protein was collected for immunoblot analysis of ZnT1. The immunoblot is representative of similar immunoblots from 3 independent experiments. **(B and C)** Light intensity was quantified for Zn- **(B)** and IGG- **(C)** treated samples and expressed relative to their respective controls. **(D)** HCT116 cells were treated with IGG, PB, TG or ZnSO_4_ for 8 hours and protein was collected for immunoblot analysis of metallothionein expression. Similar results were obtained in two independent experiments. Uncropped immunoblots are provided as S2 Fig and S3 Fig for A and D, respectively. Raw data is available in S6 File.

So far, metal responsive gene expression was presented at the RNA level only. In response to ZnSO_4_, ZnT1 protein was readily detected and protein levels increased about 3-fold (Fig 7A and 7B), comparable to mRNA levels (recall Fig 2A). In contrast, we did not detect a significant increase in ZnT1 protein levels in response to IGG (Fig 7A and 7B). We were unable to consistently detect MT expression by western blot using several antibodies or by ELISA using a commercially available kit (Abbkine, Cat: KTE61513). Therefore, MT expression was below our level of detection except in our positive control ZnSO_4_ treated samples (Fig 7D), even though we readily detected MT mRNA before and after induction by IGG (Figs 1 and 2). Thus we were unable to quantify fold changes in MT protein expression but it is clear that IGG does not strongly induce MTs or ZnT1.

**Fig 8:**
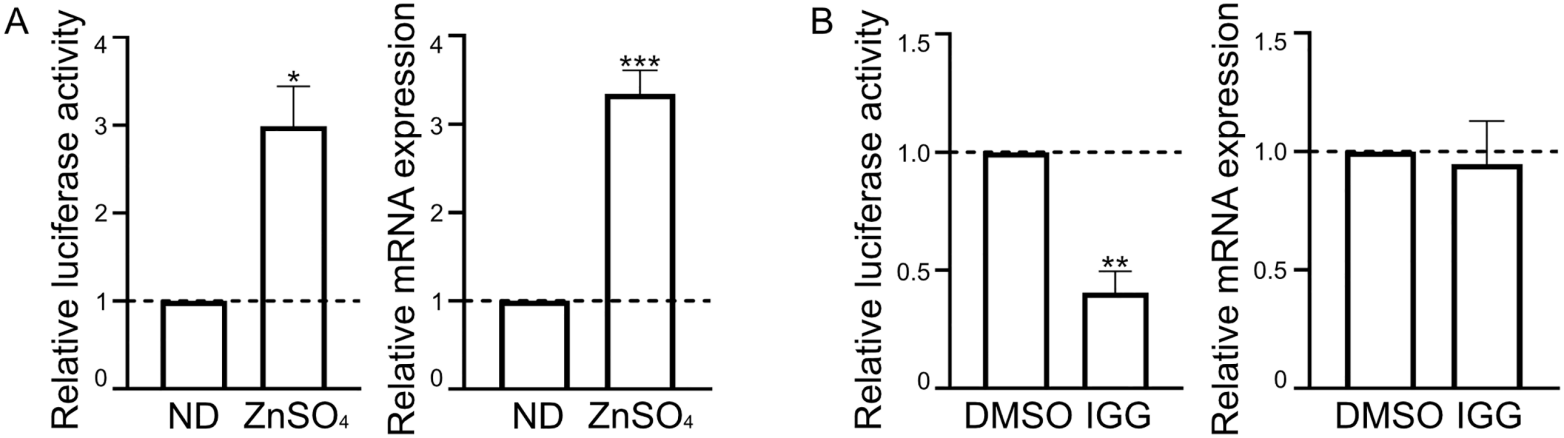
IGG inhibits a metal responsive luciferase reporter gene. U-2 OS MRE luciferase reporter cells were treated with either ZnSO_4_ **(A)** or IGG **(B)** and reporter gene activity was assessed 8 hours later. Each value represents the mean fold change (+/- SEM) relative to its corresponding control, determined from at least 3 independent experiments. *, **, and *** indicate that the value is significantly different by one sample t-test from the value 1 (P < 0.05, P < 0.01, P < 0.001). Raw data is available in S7 File.

As an alternative approach, we generated a luciferase reporter cell line from U-2 OS cells in which luciferase is driven by 5 copies of an MRE sequence (TGCRCNC where R = A or G and N is any nucleotide) to further assess the role of MTF-1 in the IGG-induced metal response. The luciferase reporter protein was engineered to contain a PEST sequence, rich in proline (P), glutamic acid (E), serine (S), and threonine (T), that decreases the half-life of the luciferase protein [16]. Reporter genes with short half-lives allow the assessment of both increases and decreases in reporter protein expression [17]. As expected, ZnSO_4_ significantly increased luciferase enzyme activity and luciferase mRNA levels (Fig 8A). In contrast, IGG did not significantly alter luciferase mRNA levels and significantly decreased luciferase activity by approximately 60% (Fig 8B). Collectively, these results suggest that the presence of multiple MRE elements in this construct was insufficient for IGG to induce reporter gene expression. Furthermore, there was a disconnect between mRNA and protein expression following IGG exposure for ZnT1, MTs, and luciferase.

**Fig 9:**
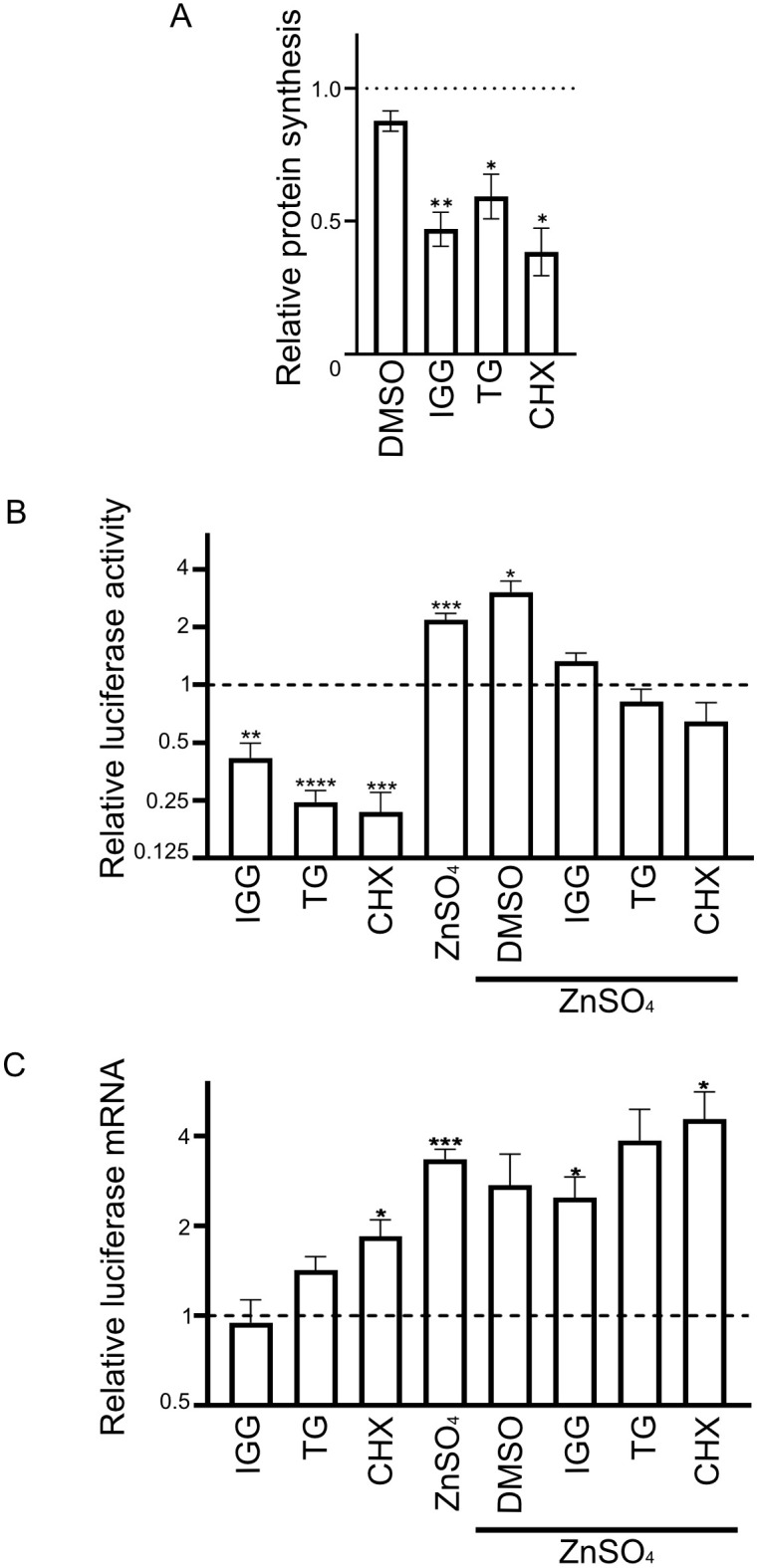
IGG, TG, and CHX attenuate ZnSO_4_-induced increase in luciferase activity. **(A)** U-2 OS MRE luciferase reporter cells were treated with IGG, Tg, or CHX for 8 hours and nascent protein synthesis was assessed using the Click-iT® HPG Alexa Fluor® Protein Synthesis Assay. **(B)** U-2 OS MRE luciferase reporter cells were treated with IGG, Tg, or CHX alone or with ZnSO_4_ for 8 hours. Luciferase activity **(B)** and mRNA expression **(C)** were assessed after 8 hours. Each value in A-C represents the mean (+ SEM) determined from at least 3 independent experiments. *, **, ***, or **** indicates that the indicated value is significantly different (P < 0.05, P < 0.01, P < 0.001 or P < 0.0001 respectively) from its controls determined by a one-sample t-test. Raw data is available in S8 File.

IGG inhibits translation and this is thought to occur through activation of the UPR and/or ISR pathways [6,8]. The fact that IGG inhibited reporter gene activity but not luciferase mRNA expression suggests that IGG could be inhibiting translation of the luciferase reporter gene. To test this, we treated cells with ZnSO_4_ alone or in combination with IGG, Tg (an activator of the UPR) [18], or cycloheximide (a protein synthesis inhibitor) [19]. Individually IGG, Tg and CHX each inhibited nascent protein synthesis and luciferase activity (Fig 9A and 9B). In contrast, ZnSO_4_ increased reporter gene activity approximately 2.5- to 3-fold (Fig 8A). Consistent with their effects on translation, IGG, Tg and CHX prevented the full increase in luciferase activity elicited by ZnSO_4_ (Fig 9B). Importantly, this inhibitory effect of IGG, Tg and CHX on ZnSO_4_-induced luciferase activity was not detected at the mRNA level (Fig 9B). Our results suggest that IGG, Tg and CHX prevent the translation of the metal responsive luciferase mRNA.

## Discussion

IGG was first reported in the literature in the early 1980’s as one of several biflavones isolated from *G. biloba* leaf extracts [20]. It was later found to inhibit cell growth in culture and induce apoptosis in a variety of cell lines [21–23]. The precise target of IGG contributing to these biological effects has yet to be determined but, IGG was identified in a chemical screen as a potent small molecule inhibitor of the spliceosome [24]. IGG inhibits the recruitment of the U4/U5/U6-tri snRNP complex resulting in a failure to progress from the A to the B complex of the spliceosome [24]. A failure to properly splice pre-mRNAs could lead to 1. A failure to express functional proteins, 2. The accumulation of abnormal RNAs, and/or 3. The production of proteins with altered function [5]. It is conceivable that some of the biological effects of IGG could be related to spliceosome dysfunction.

To investigate the effect of spliceosome dysfunction on cell cycle and apoptosis, we previously compared IGG responses to those of PB, an SF3B1 inhibitor that interferes with spliceosome assembly at an earlier stage [23,25]. First, we found considerable similarity in cell cycle responses. IGG and PB both delayed passage of cells through G_1_, S and G_2_, but not M phase, with minor differences in the relative contribution of changes to these cell cycle phases [25]. Therefore, these spliceosome inhibitors have similar effects on cell cycle progression.

Additional similarity in cellular responses to IGG and PB was detected through genetic analysis of apoptosis induced by these compounds [23]. Briefly, transcriptome analysis of IGG treated cells identified the ATF4 transcription factor as a central player in the IGG transcriptional response [6,8]. This is consistent with the PERK-dependent branch of the UPR and ISR [6,8]. The PERK/eIF2α/ATF4 pathway is generally adaptive in response to ER stress although prolonged ER stress can lead to ATF4-dependent cell death [10,26,27]. Activating transcription factor 3 (ATF3) is another Basic Leucine Zipper (bZIP) transcription factor that can be directly regulated by ATF4, placing ATF3 downstream of ATF4 in the UPR and ISR [28–31]. Importantly, ATF3 contributes to apoptosis in turn by regulating proapoptotic proteins like CHOP, and CHAC1 [32,33] that are reported to be increased in an ATF4-dependent manner following exposure to IGG [6,8]. The ATF3 transcription factor is induced by both compounds at cytotoxic concentrations, and consistent with a role of ATF3 in promoting cell death, deletion and knockdown of ATF3 protected human and murine cells from this form of cell death [23]. Therefore, ATF3 also plays a key role in the cellular response to these splicing inhibitors.

In our transcriptome analysis of the IGG response, we detected another less prominent signature that resembled the RMI [6,8] Here, we characterized this response to IGG and found that IGG induces metallothionines and a zinc transporter at the mRNA level. The expression of these transcripts was dependent on MTF-1 prior to and following IGG exposure, consistent with the RMI. Using a metal responsive reporter system containing 5 copies of a single specific MTF-1 binding site (TGCACAC) driving luciferase expression, we found that IGG did not increase mRNA levels while our positive control ZnSO_4_ did. Therefore, the presence of the MRE sequence was not sufficient for IGG to increase reporter mRNA expression, under these conditions. This could result from the fact that IGG does not induce RMI as strongly as ZnSO_4_ (recall Figs 1 and 2). It should also be pointed out that the MRE consensus sequence is TGCRCNC where R = A or G and N is any nucleotide [34]. Thus, the MRE luciferase reporter only contains one specific version of the MTF-1 binding site. Notably, the response of specific metal responsive genes varies by metal ion and by ion concentration [35–38]. It is possible that this specific reporter is not optimal to detect the IGG-induced RMI transcriptional response.

ZnSO_4_ also increased luciferase activity in the reporter cell lines, while IGG, Tg and CHX all decreased luciferase activity. This decrease in luciferase activity is consistent with the fact that IGG, Tg and CHX inhibit nascent protein synthesis [8,19,39]. To assess this further, we co-treated cells with ZnSO_4_ and IGG, Tg, or CHX to determine if they could block the ZnSO_4_ response at the RNA and protein level. IGG, Tg and CHX inhibited ZnSO_4_ induced luciferase activity with CHX being most effective inhibitor and IGG the least effective. Importantly, none of these compounds prevented the ZnSO_4_-induced increase in luciferase mRNA. Taken together, the dissociation between mRNA and protein expression in IGG treated samples is likely due to the fact that IGG indirectly inhibits nascent protein synthesis [8]. So, although the RMI seems to be activated by IGG, there is no evidence that there is a functional consequence to this transcriptional response, as least under the conditions tested.

How IGG activates the RMI remains unclear. There are a variety of possibilities and work is ongoing to determine the precise mechanism. As indicated earlier, IGG is reported to be a spliceosome inhibitor [24]. It is possible that metal responsive genes respond to some form of splicing stress response. MTF-1 is a zinc finger protein whose subcellular localization and binding to MREs is controlled by zinc. It is not clear if the IGG induced RMI is affected by zinc levels or if IGG directly or indirectly affects intracellular zinc. Notably, there are other *cis*-acting elements in the MT promoter and enhancer regions including antioxidant response elements. Not surprisingly, MT expression can be increased in response to oxidative stress [40–42]. In the present work, the MRE sequences in the reporter cell line were insufficient for IGG to increase luciferase expression, despite the importance of MTF-1 in the IGG induced RMI. It is conceivable that IGG contributes to the metal response through oxidative stress instead of heavy metals. Our ongoing efforts will be required to test these possibilities.

## Conclusions

Existing research on cellular responses to IGG have revealed the broad cellular activity and potential anti-neoplastic effects of IGG, though detailed mechanistic responses to IGG and direct consequences of its splicing inhibitory activity are not fully defined. Here, we further established that IGG induces the expression of a group of MTs and ZnT1 involved in the RMI at the mRNA but not protein level, independent of ATF4. Treatment with the ER stressor, Tg, also appeared to increase expression of these metal responsive transcripts, raising the possibility that IGG may be inducing the RMI through some aspect of ER stress that does not involve ATF4. This implies that ER stress may activate the RMI but that this is simultaneously antagonized by the effects of ER stress on translation.

## Supporting information

S1 Fig Uncropped immunblot images presented in Fig 3E. Here the membrane was cut in half and the upper and lower halves were probed with antibodies to MTF-1 and actin, respectively.
(TIFF)

S2 Fig Uncropped immunblot images presented in Fig 7A. Here the blot was probed with an antibody recognizing ZnT1 (left) and subsequently reprobed with the anti-actin antibody (right). The bands denoted by * in the actin blot represent those that remain visible from the ZnT1 western.
(TIFF)

S3 Fig Uncropped immunoblot images presented in Fig 7D. Here proteins were separated on a 16% polyacrylamide gel. The blots were probed first with anti-MT antibody (upper panel) and then anti-actin antibody (lower panel). The MT band remained visible following immunoblotting with anti-actin.
(TIFF)

S1 File Data summarized in Fig 1. (XLSX)

S2 File Data summarized in Fig 2. (XLSX)

S3 File Data summarized in Fig 3. (XLSX)

S4 File Data summarized in Fig 4. (XLSX)

S5 File Data summarized in Fig 6. (XLSX)

S6 File Data summarized in Fig 7. (XLSX)

S7 File Data summarized in Fig 8. (XLSX)

S8 File Data summarized in Fig 9. (XLSX)
