# Supplementary figures and images for: Isoginkgetin increases the expression of metal responsive transcripts but inhibits their translation
Source: PLoS One. 2026 Jun 25;21(6):e0352014. doi: 10.1371/journal.pone.0352014 (PMC13298952; doi:10.1371/journal.pone.0352014)

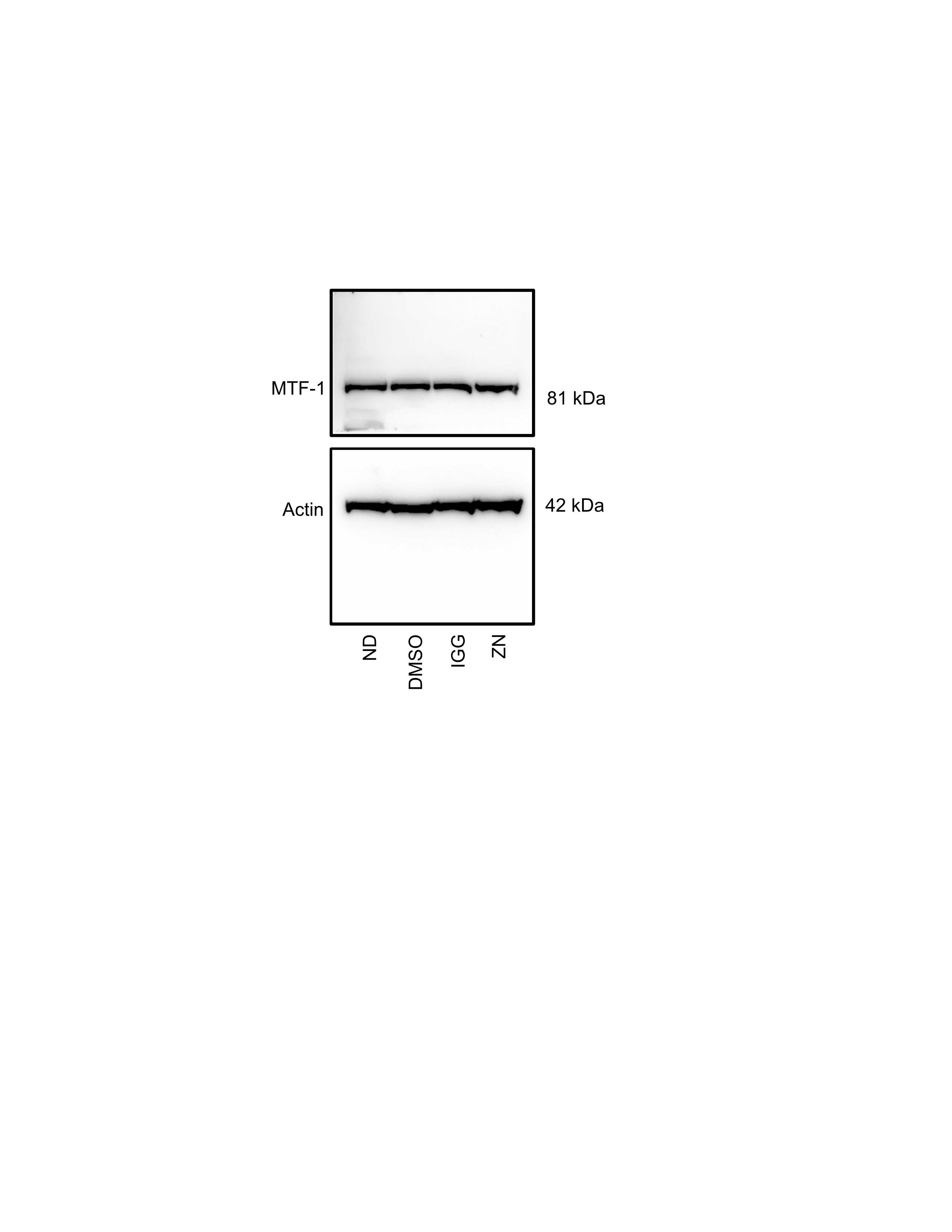

Supplement: S1 Fig — Here the membrane was cut in half and the upper and lower halves were probed with antibodies to MTF-1 and actin, respectively. (TIFF) [file pone.0352014.s001.tiff]

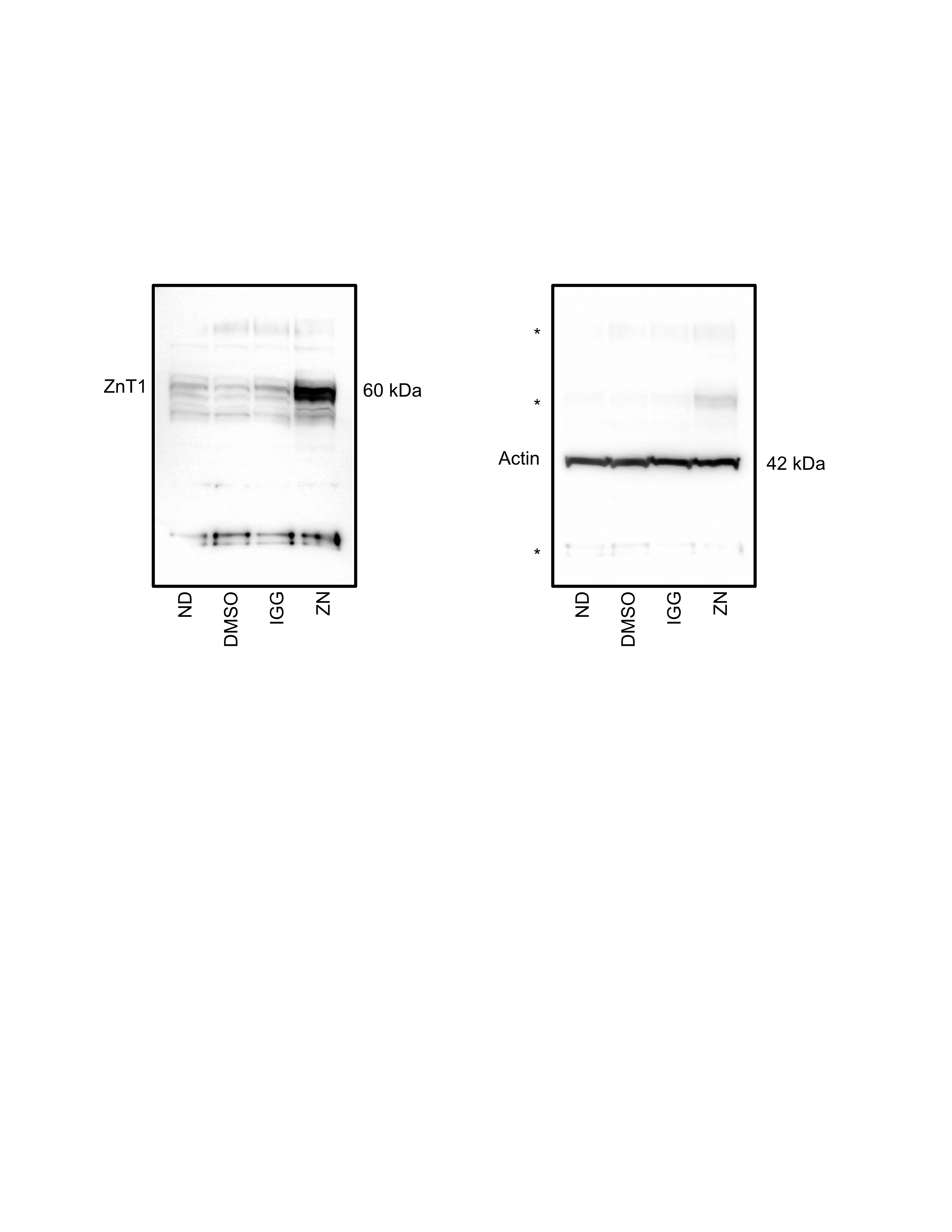

Supplement: S2 Fig — Here the blot was probed with an antibody recognizing ZnT1 (left) and subsequently reprobed with the anti-actin antibody (right). The bands denoted by * in the actin blot represent those that remain visible from the ZnT1 western. (TIFF) [file pone.0352014.s002.tiff]

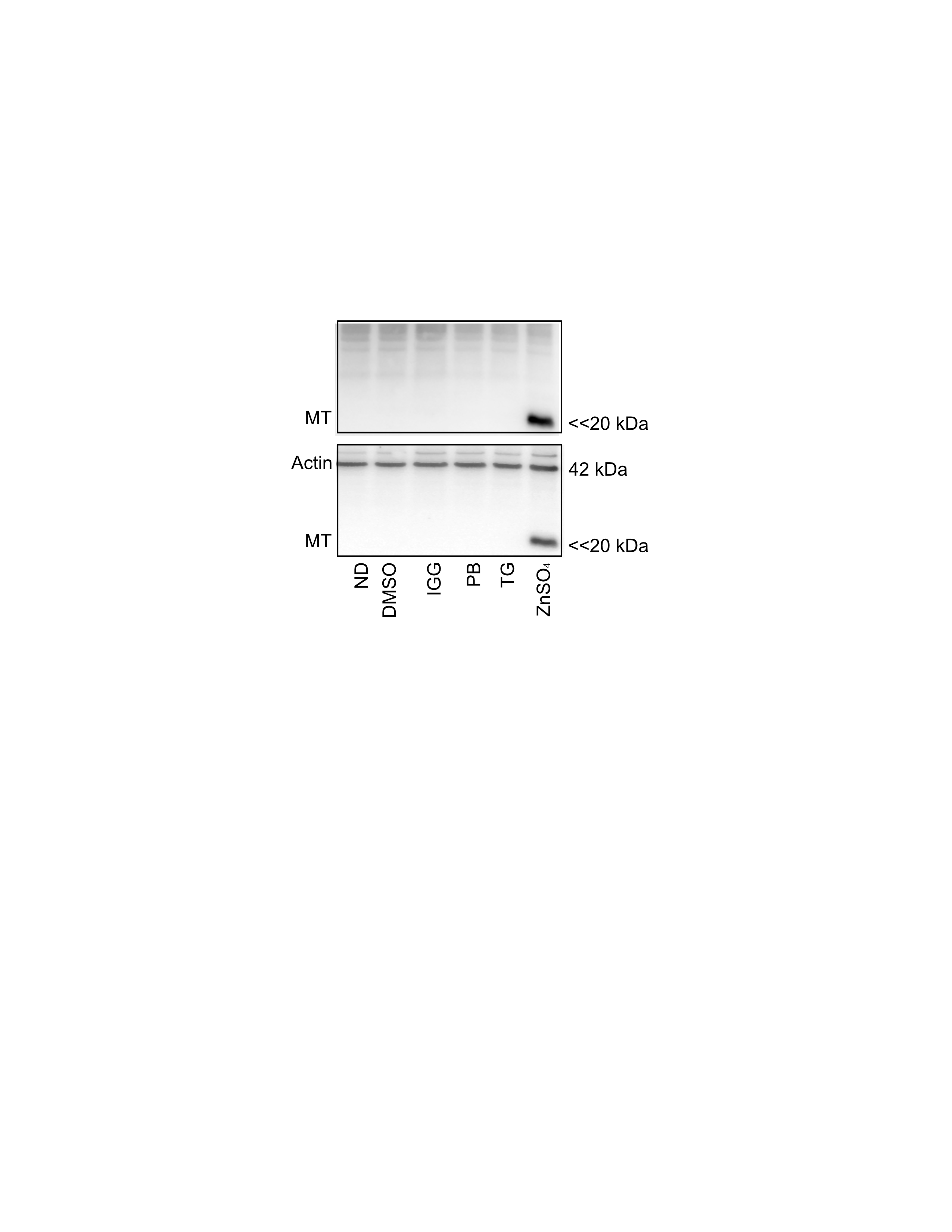

Supplement: S3 Fig — Here proteins were separated on a 16% polyacrylamide gel. The blots were probed first with anti-MT antibody (upper panel) and then anti-actin antibody (lower panel). The MT band remained visible following immunoblotting with anti-actin. (TIFF) [file pone.0352014.s003.tiff]
